# eTumorType, An Algorithm of Discriminating Cancer Types for Circulating Tumor Cells or Cell-free DNAs in Blood

**DOI:** 10.1016/j.gpb.2017.01.004

**Published:** 2017-04-04

**Authors:** Jinfeng Zou, Edwin Wang

**Affiliations:** 1 National Research Council Canada, Montreal, QC H4P 2R2, Canada; 2 Department of Experimental Medicine, McGill University, Montreal, QC H3A 2B2, Canada; 3 Center for Bioinformatics, McGill University, Montreal, QC H3G 0B1, Canada; 4 Center for Health Genomics and Informatics, University of Calgary Cumming School of Medicine, Calgary, AB T2N 4N1, Canada; 5 Department of Biochemistry & Molecular Biology, University of Calgary Cumming School of Medicine, Calgary, AB T2N 4N1, Canada; 6 Department of Medical Genetics, University of Calgary Cumming School of Medicine, Calgary, AB T2N 4N1, Canada; 7 Department of Oncology, University of Calgary Cumming School of Medicine, Calgary, AB T2N 4N1, Canada; 8 Alberta Children’s Hospital Research Institute, Calgary, AB T2N 4N1, Canada; 9 Arnie Charbonneau Cancer Research Institute, Calgary, AB T2N 4N1, Canada; 10 O’Brien Institute for Public Health, Calgary, AB T2N 4N1, Canada

**Keywords:** eTumorType, Founding clone, Copy number variation, Non-invasive detection, Cancer

## Abstract

With the technology development on detecting circulating tumor cells (CTCs) and cell-free DNAs (cfDNAs) in blood, serum, and plasma, non-invasive diagnosis of **cancer** becomes promising. A few studies reported good correlations between signals from tumor tissues and CTCs or cfDNAs, making it possible to detect cancers using CTCs and cfDNAs. However, the detection cannot tell which cancer types the person has. To meet these challenges, we developed an algorithm, **eTumorType**, to identify cancer types based on **copy number variations** (CNVs) of the cancer **founding clone**. **eTumorType** integrates cancer hallmark concepts and a few computational techniques such as stochastic gradient boosting, voting, centroid, and leading patterns. **eTumorType** has been trained and validated on a large dataset including 18 common cancer types and 5327 tumor samples. **eTumorType** produced high accuracies (0.86–0.96) and high recall rates (0.79–0.92) for predicting colon, brain, prostate, and kidney cancers. In addition, relatively high accuracies (0.78–0.92) and recall rates (0.58–0.95) have also been achieved for predicting ovarian, breast luminal, lung, endometrial, stomach, head and neck, leukemia, and skin cancers. These results suggest that **eTumorType** could be used for non-invasive diagnosis to determine cancer types based on CNVs of CTCs and cfDNAs.

## Introduction

Non-invasive detection of cancer is becoming possible with the development of technologies on capturing circulating tumor cells (CTCs) and cell-free DNAs (cfDNAs) in blood, serum, and plasma [1], [2], [3]. However, it is still challenging to get accurate abnormal signals on cancer genomes, as CTCs and cfDNAs are rare in blood samples. For example, there are about 1–100 CTC(s) per 109 blood cells [4], resulting in the generation of false negatives, false positives, or biased signals due to the amplification procedure [5], [6]. Moreover, hundreds of depths of a cancer genome are usually needed to achieve good accuracy and coverage for mutation calling [7], causing difficulty in getting a large amount of data for early-stage patients which would need even more sequencing depth to capture signals reliably. Thus, copy number variation (CNV) attracts more attention, because CNV signals could be detected with relatively low-depth sequencing of samples. Furthermore, CNV detection has a low burden in cost because CNVs usually involve a segment of chromosomes with relatively strong intensity, especially for amplification [2]. Therefore, it is very promising to diagnose and detect cancer at early stages based on CNVs. Technologies for CTC cell capture is being developed. We could easily detect and capture CTCs in blood samples even for people without diagnosis of cancer, but might not determine cancer types just based on CTC cell biology, because CTCs are usually identified insensitively by isolating non-blood cells and epithelial cells for solid cancers. Cancer type could be potentially determined based on the genomic information of the CTCs, which would help the clinicians to decide the proper organs of cancer patients for further check. Therefore, we developed a novel algorithm, eTumorType, to determine cancer types based on the CNVs of tumors, which could be applied on the data of CTCs.

It is well known that there are multiple clones within a tumor, including a founding clone and several sub-clones [8], [9]. The founding clone is the most recent founder cancer cell of a tumor. All the genomic changes such as somatic mutations and CNVs that occur in the founding clone will be carried on in all the cancer cells of that tumor. Thus, CNVs of a founding clone will be found in CTCs as well. Therefore, the analysis based on the CNVs of cancer founding clone would reflect the result on CTC genomic data. Thus, eTumorType is designed to model the CNVs in cancer founding clones. As a popular tool for evaluating the purity and ploidy of tumor cells, ABSOLUTE identifies somatic CNV segments belonging to the founding clone and sub-clones as well using CNVs [10]. Furthermore, a primary feature of cancer is proliferation of cancer cells, involving a multitude of highly-regulated oncogenes, to which genomic amplification contributes greatly [11]. Moreover, the genomic amplification is more likely to be detected than deletion as genes only have two copies to lose whereas could reach 4.4 copies and more by amplification [12]. Apparently, amplification signals would be easier and more accurately identified. Therefore eTumorType will focus on modeling of the genomic amplifications.

Robustness is always the most important consideration in developing algorithms. To deal with this issue, methods based on the stochastic mechanisms have been developed. For example, multiple survival screening (MSS) has been developed to achieve robustness by screening random gene sets and random datasets and then selecting genes with higher probability of contributing to robustness. In cancer samples, these genes are often cancer hallmark-associated genes [13], [14]. However, MSS has been proposed and validated using gene expression data with continuous values. Random forest (RF) is a popular ensemble method of constructing decision trees using bootstrap samples and random features, and then classifying with a strategy based on these trees [15]. ada is also an ensemble method, which integrates the stochastic gradient boosting with a stochastic mechanism and refinement on the training set in each boosting step, thus able to generate the ensemble at a higher speed [16]. Both RF and ada are suitable to analyze continuous data and discrete values.

In this study, we developed a computational algorithm, eTumorType, by modeling CNVs in the founding clone (*i.e.*, genomic amplifications) of the cancer hallmark-associated genes (*i.e.*, one of the key factors for reaching robustness in MSS) using ada, considering that CNVs are often presented as discrete values and ada shows advantage in speed using the stochastic gradient boosting procedure. Furthermore, we also applied a combinatory signature set approach in eTumorType [13], [14]. Multiple cancer hallmark-derived models were employed and the centroid of the number of them supporting each cancer type prediction was generated and then used for predicting cancer types for a given sample based on the correlation coefficient between them. Finally, a leading pattern-weighted correlation method was developed in the eTumorType. eTumorType was validated using 2133 (40% of 5327) samples, indicating that it is able to successfully discriminate 14 out of 18 common cancer types with high accuracy and power (recall rate). We hope that this tool could be used for cancer diagnosis based on the CNVs of captured CTCs or cfDNAs in blood samples in the future.

## Method

### SNP data

SNP 6.0 microarray data of tumors for 18 cancer types were collected from The Cancer Genome Atlas (TCGA) database (Table 1). Given their genetic differences, the luminal and basal subtypes of breast cancer were treated as different cancer types. On the other hand, colon and rectum cancers were integrated together as one cancer type, since they have similar genomic profiles [12].

### Detection of somatic CNVs

The segmentation files annotated based on the reference genome of hg19 were downloaded from TCGA. These files were used as inputs to the GISTIC 2.0 [17] in the GenePattern online platform [18]. The output of the software is a gene-based GISTIC score profile, which was used to evaluate the copy number changes for genes. A gene is defined as amplified with the GISTIC score >0.3, [19]; otherwise, this gene is defined as a non-amplified. We used 1 and 0 to represent amplified and non-amplified, respectively, for genes in the CNV profile of each sample.

### Identification of somatic founding clone CNVs

ABSOLUTE is used to identify aberrational chromosome segments involved in the founding clone or sub-clones in the tumor tissues [10]. Of note, because ABSOLUTE may fail to detect CNVs in some samples due to the poor quality of data, we only retained the samples which have CNV profiles generated by ABSOLUTE. The CNV profiles were filtered by the output from ABSOLUTE to generate the somatic founding clone amplification profiles, which were used for the subsequent analysis. The dataset was randomly split into three subsets with proportions as 60%, 20%, and 20% for the training set, the validation set, and the test sets, respectively. The cancer types and the corresponding sample sizes are summarized in Table 1.

### The eTumorType algorithm

**Figure 1:**
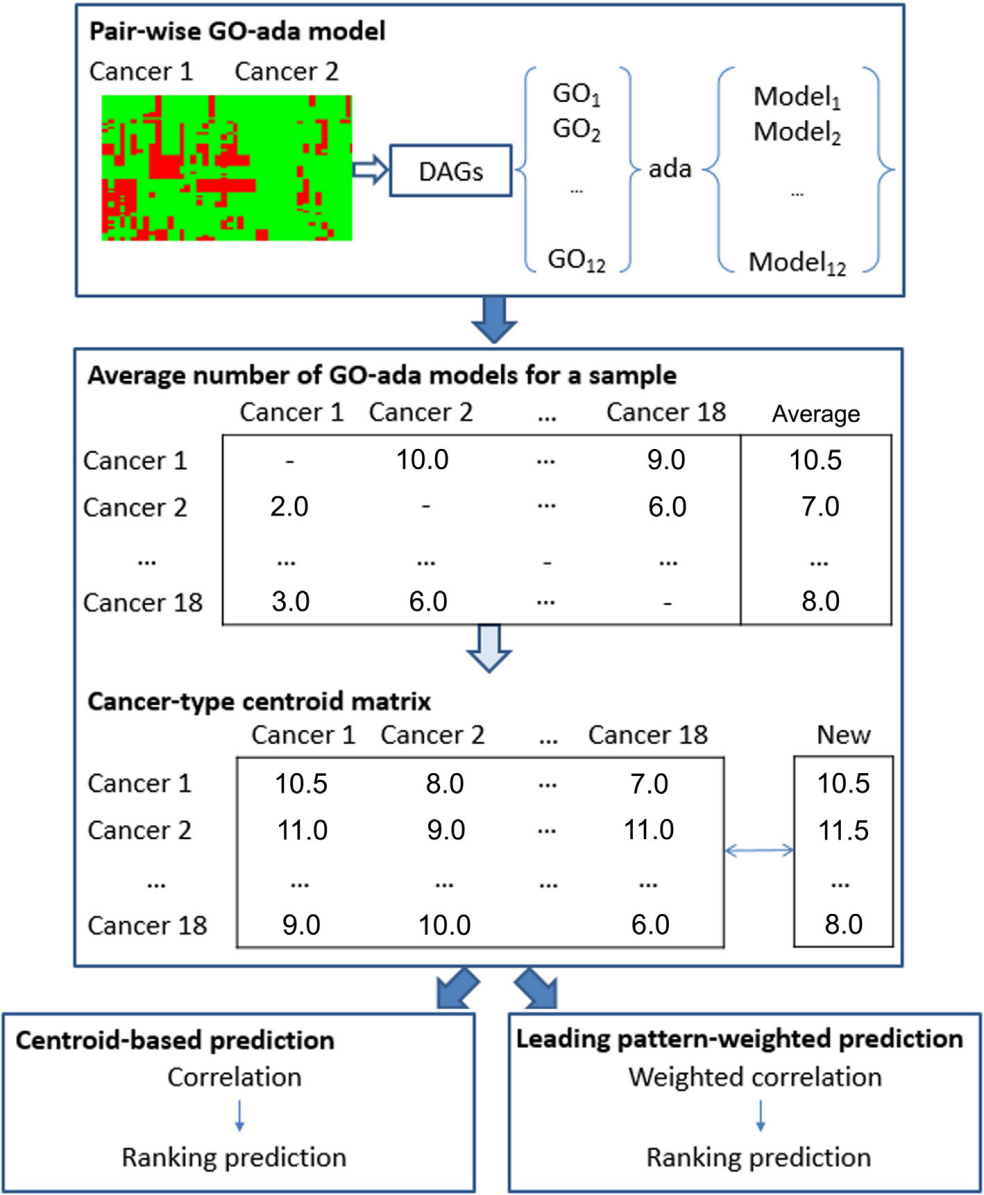
**Scheme of the eTumorType algorithm** Pair-wise GO-ada model: the CNV profiles (rows for genes and columns for samples) for cancer type 1 (cancer 1) and cancer type 2 (cancer 2) were used to select significant DAGs. DAGs associated with six cancer hallmarks as annotated with GO terms were retained and 12 GO sets were selected (see Method) and input into ada R package to build GO-ada models. Average number of GO-ada models for a sample: for a given sample, the numbers of GO-ada models favoring each cancer type prediction were constructed as a matrix based on all the 12 × $C_{18}^{2}$ models and then the vector of average number of GO-ada models was created. Next, cancer-type centroid matrix was built by collecting the centroid vector of average number of GO-ada models for all the 18 possible cancer types (the rows of the matrix) on each cancer type (the column of the matrix) data of the training set. Centroid-based prediction: for a given new sample, the vector of the average number of GO-ada models favoring each cancer type prediction was calculated and then used for evaluating its correlations with the centroid vector of each cancer type (column of the cancer-type centroid matrix). The correlation coefficients were ranked and the 3 top-ranked cancer types were selected as the final prediction. Leading pattern-weighted prediction: the same procedure as the centroid-based prediction was performed except that the weighted correlation replaced the simple correlation (see Method). GO, Gene Ontology; CNV, copy number variation; DAG, differentially-amplified gene.

There are three layers in the eTumorType algorithm (Figure 1): (1) building cancer-pair-wise gene ontology (GO) ada models (GO-ada models) based on 12 cancer hallmark-associated GO terms, each of which contains a set of discriminating amplified cancer hallmark-associated genes; (2) voting cancer type of either one or another using the GO-ada models, getting the centroid of the number of GO-ada models voting for each cancer type prediction, and then making a centroid-based correlation prediction; and (3) finally, making a leading pattern-weighted correlation prediction.

### Constructing pair-wise cancer hallmark-based ada models

The cancer hallmark-associated genes were collected based on GO annotations of genes for cancer hallmarks [20], [21], [22]. In this study, six cancer hallmarks were selected: apoptosis, cell adhesion, cell cycle, cell proliferation, phosphorylation, and immune response.

Significant differentially amplified genes (DAGs) between any two cancer types were identified using fuzzy analysis clustering with R package on the training set. For a given pair of cancer types, the DAGs of each selected cancer hallmark were used to construct ada predictive models for discriminating cancer types. Considering the effect of sample size on the statistical significance and the expectation of sufficient DAGs, a composite approach was employed: (1) if the sample sizes of both cancer types are smaller than 200, a loose *P* value of 0.01 based on Fisher’s exact test was set for statistical significance; (2) if the sample size of one cancer type is smaller than 200, but that of the other cancer type is larger than 200, the *P* value threshold was set to 0.005; (3) if the sample sizes of both cancer types are larger than 200, the *P* value cut-off was set to 0.001. Here the number of 200 was chosen considering the sample size distribution of the training sets for the 18 cancer types examined. For the eleven, three, and four cancer types, each contains >200, <100, and 100–200 samples, respectively. The DAGs were grouped based on cancer hallmarks they are belonging to. If a gene belongs to multiple cancer hallmarks, it was assigned to multiple gene groups. The selected genes annotated in the six cancer-hallmark GO terms were retained for the subsequent analysis. For each GO term, we ranked its genes based on the product of amplification degree and amplification difference between two cancer types (*i.e.*, gene A ranks higher than gene B if the score of A is higher than that of B). The top-30 and top-100 genes were tried for constructing models that classify the two cancer types using the ada R package [16]; the models are denoted as GO-ada models. We reported the results using the top-100 genes in this manuscript. Finally, 12 ada models were constructed for discriminating each pair of cancer types.

### A centroid-based correlation prediction

This part contains voting cancer types of either one or another using GO-ada models, and getting the centroid of the number of GO-ada models voting for each cancer type. The procedure of building GO-ada models based on 12 GO terms produced 12 GO-ada models for each pair of cancer types, and thereby 12 × $C_{18}^{2}$ models were created in total (Figure 1). For a given sample, a prediction matrix (18 × 18) was generated by these models, which is composed of the number of GO-ada models predicting the sample as the cancer types listed in the rows. For example, for the comparison between cancer type 1 and cancer type 2, 10 of the 12 GO-ada models between them predicted the sample to be cancer type 1, while the other two models predicted it to be cancer type 2 (Figure 1). Then, a vector of the average numbers of GO-ada models for all possible cancer types was generated alongside the rows of the matrix. The rankings of cancer types in the vector indicate their probability that the cancer type a sample could be considered as. That is, the higher average number of GO-ada models voting for a particular cancer type when compared with the other 17 cancer types, the higher probability the cancer type being this particular cancer type. Next, a matrix was created by collecting the vectors of all training samples belonging to a cancer type, which is composed of 18 possible cancer types and samples. Thereafter, a centroid vector for all possible cancer types on the matrix was generated by calculating the centroid of average number of GO-ada models for each row of the matrix using the pamr R package (https://cran.r-project.org/web/packages/pamr/index.html). Finally, a cancer-type centroid matrix was created by pooling the centroid vectors for all cancer types together, which is 18 possible cancer types × 18 cancer types included in the training set (Figure 1).

For the prediction of a new sample, the vector of average number of GO-ada models voting for each possible cancer type was firstly created based on the predictions using all models (the method is mentioned above). Secondly, the correlation coefficient between it and the centroid for each cancer type (the cancer-type centroid matrix) was calculated. Thirdly, the correlation scores for all possible cancer types were ranked; a higher ranking suggests a higher probability that the given sample might belong to that cancer type (Figure 1). In this study, a strategy of top-ranked candidate set was proposed to improve the reliability of cancer type prediction. We took 1–3 top-ranked cancer types as a final prediction for the given sample. For example, for a luminal breast cancer sample, if the luminal breast cancer type was included in the top-3 cancer types, we considered the prediction to be correct. The procedure of evaluating accuracy and power (recall rate) was: (1) if the true cancer type for a sample is included in the top-ranked candidate set, the true cancer type was assigned to the sample; if not, the top-1 cancer type would be taken; and (2) the accuracy and power for each cancer type prediction was calculated as # (true predictions)/ # (all predictions) and # (true predictions)/ # (true cancer type samples).

### Leading pattern-weighted correlation prediction

**Supplementary Fig. S1:**
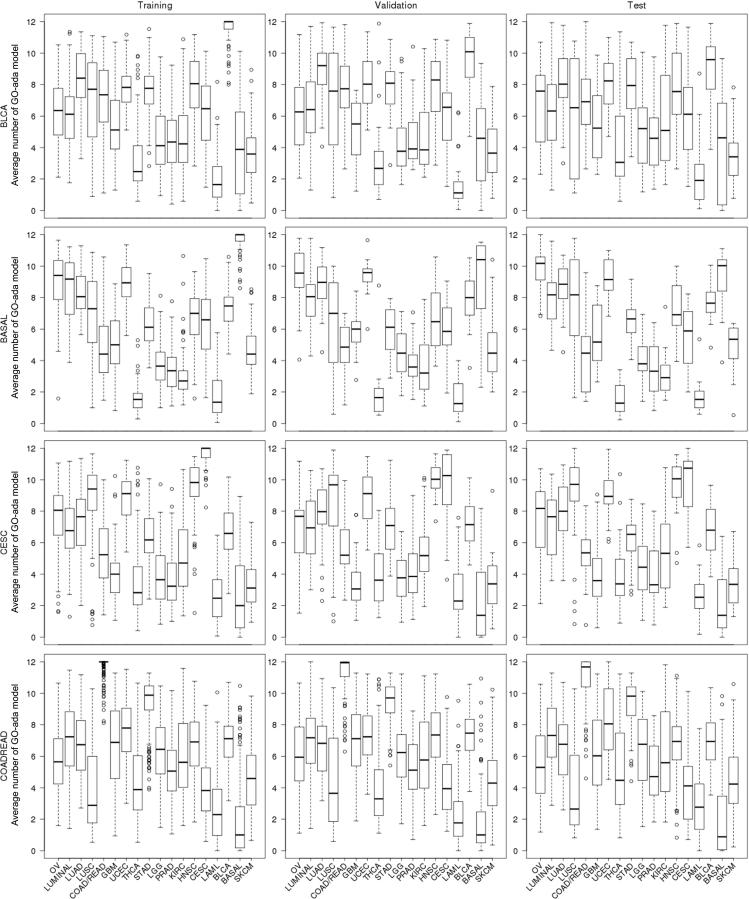
The average number of GO-ada models voting for possible cancer types for BLCA, BASAL, CESC, and COAD/READ datasets The average numbers of GO-ada models voting for possible cancer types are shown by the boxplots. The box displays the range of 25^th^ percentile and 75^th^ percentile. The circles represent the values lower than 10^th^ percentile or greater than 90^th^ percentile. The abbreviations of cancers are explained in Table 1.

**Supplementary Fig. S2:**
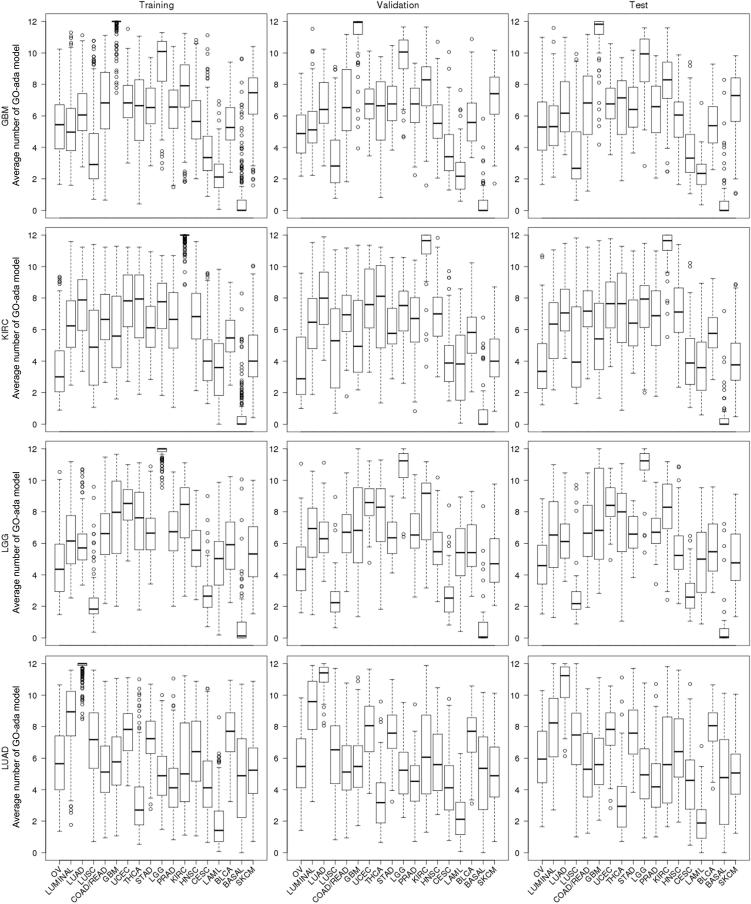
The average number of GO-ada models voting for possible cancer types for GBM, KIRC, LGG, and LUAD datasets The average numbers of GO-ada models voting for possible cancer types are shown by the boxplots. The box displays the range of 25^th^ percentile and 75^th^ percentile. The circles represent the values lower than 10^th^ percentile or greater than 90^th^ percentile. The abbreviations of cancers are explained in Table 1.

**Supplementary Fig. S3:**
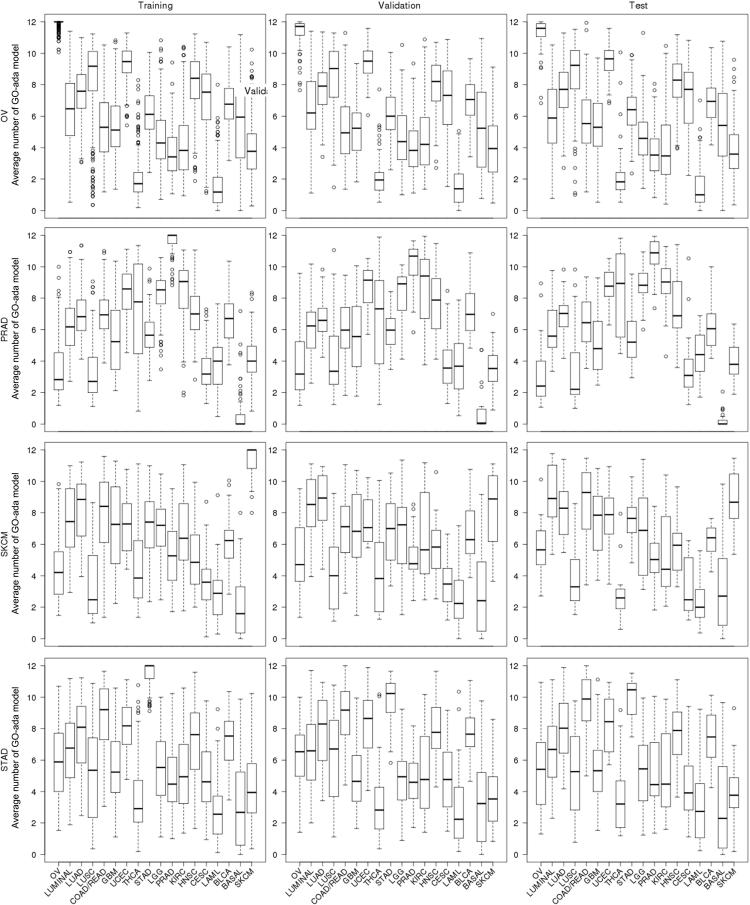
The average number of GO-ada models voting for possible cancer types for OV, PRAD, SKCM, and STAD datasets The average numbers of GO-ada models voting for possible cancer types are shown by the boxplots. The box displays the range of 25^th^ percentile and 75^th^ percentile. The circles represent the values lower than 10^th^ percentile or greater than 90^th^ percentile. The abbreviations of cancers are explained in Table 1.

**Supplementary Fig. S4:**
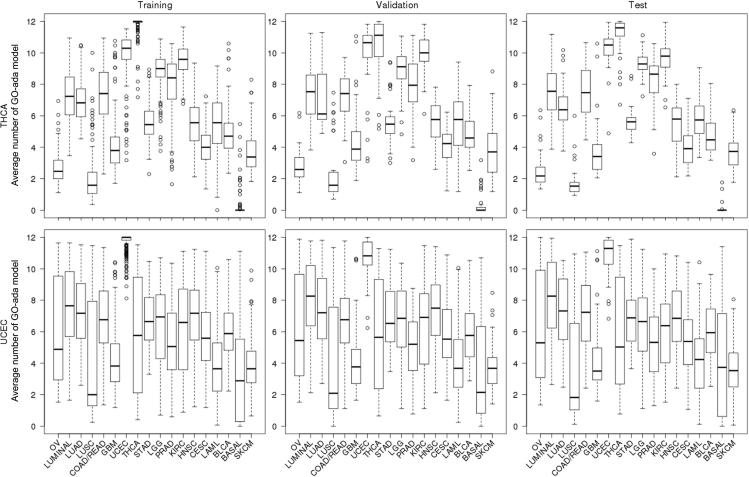
The average number of GO-ada models voting for possible cancer types for THCA and UCEC datasets The average numbers of GO-ada models voting for possible cancer types are shown by the boxplots. The box displays the range of 25^th^ percentile and 75^th^ percentile. The circles represent the values lower than 10^th^ percentile or greater than 90^th^ percentile. The abbreviations of cancers are explained in Table 1.

**Figure 2:**
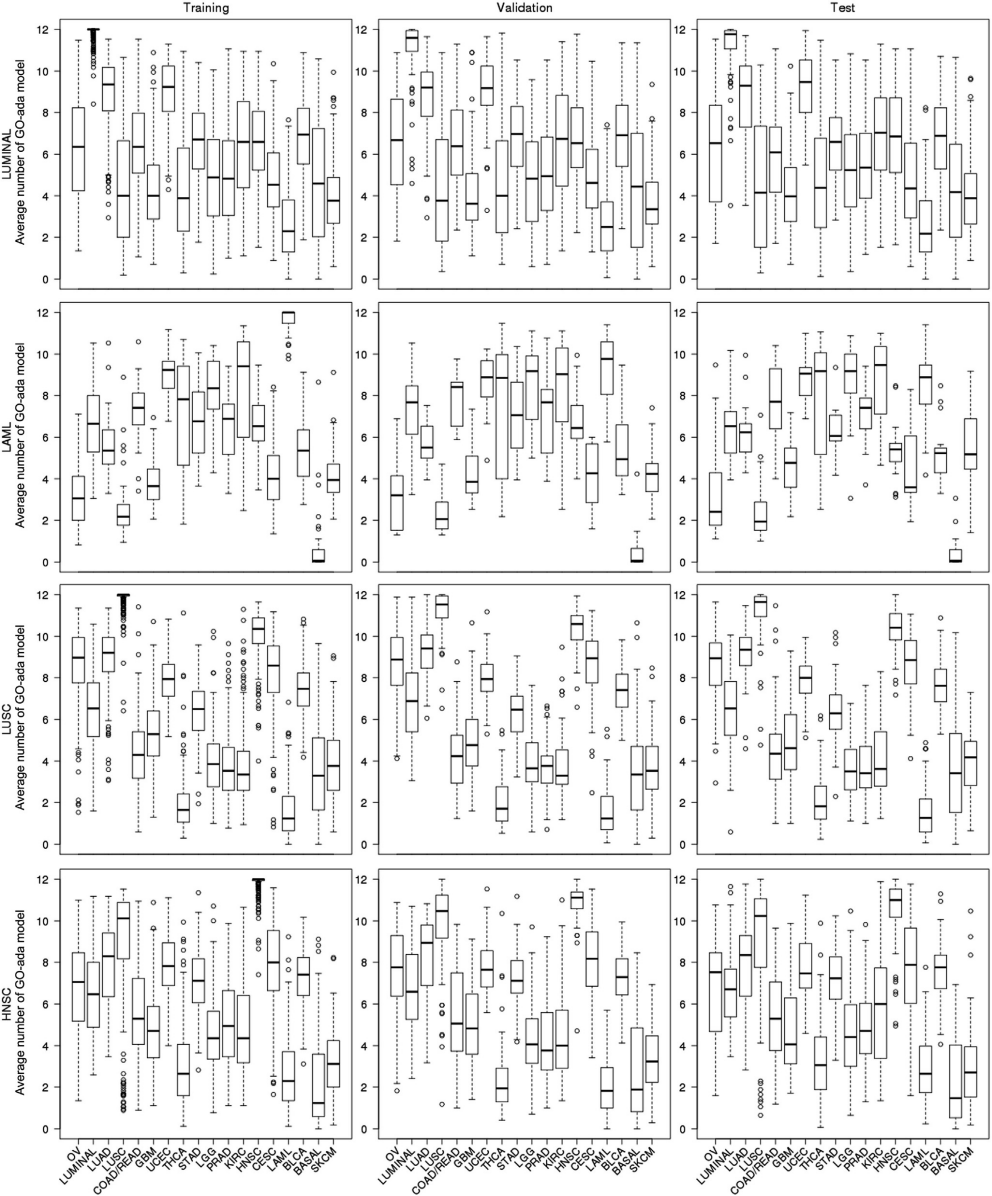
**The average number of GO-ada models voting for possible cancer types for LUMINAL, LAML, LUSC, and HNSC datasets** The average numbers of GO-ada models voting for possible cancer types are shown by the boxplots. The box displays the range of 25th percentile and 75th percentile. The circles represent the values lower than 10th percentile or greater than 90th percentile. The abbreviations of cancers are explained in Table 1.

In the training set, the average number of GO-ada models voting for a cancer type is likely to be high for some of the cancer types but low for some other cancer types (Figures 2; S1–S4). For example, lung squamous cell carcinoma (LUSC) samples got high average numbers of GO-ada model voting for ovarian serous cystadenocarcinoma (OV), lung adenocarcinoma (LUAD), head and neck squamous cell carcinoma (HNSC), and LUSC itself, but very low votes for thyroid carcinoma (THCA) and acute myeloid leukemia (LAML) (Figure 2). We took this information to improve the prediction performance. First, for each cancer type in the training set, a clustering analysis of all 18 cancer types was performed using the cluster R package (pam function) based on the matrix composed of the average numbers of GO-ada models voting for each cancer type for all samples. The clustering analysis could group the cancer types into clusters based on criterion of similar average number of GO-based models. Then, the average number of GO-ada models was estimated for each cluster. Next, two clusters with the highest and lowest average numbers were taken as the most similar and the most dissimilar cancer type groups, respectively. In order to limit the influence of taking the similarity and dissimilarity among cancer types for the prediction, the numbers of possible cancer types for the two categories need to be controlled. For details, based on the matrix mentioned above for each cancer type, we screened a set of pre-defined 3, 4, 5, and more clusters for evaluating the sizes of the leading up-/bottom-clusters. Based on the results, the maximum numbers of 5 and 3 for the leading up-/bottom-clusters, respectively, were used in this study (Table S1). We also observed that changing the sizes (maximum numbers) slightly to 4 and 2 did not change accuracy and power much.

Next, the weighted correlation analysis was performed. Briefly, a high (2, 3, 4, and 5) and low (0.1, 0.2, and 0.3) weights were assigned to leading up-/bottom-patterns, respectively; the weight of 1 was set for the other cancer types. The setting of 3 for the leading up-patterns showed a relatively better performance on accuracy and power in the validation set (the final validation was done in the test set). For the leading bottom-patterns, the performances for the various settings were similar. The results reported in this study were based on the weights of 3 and 0.1 for the leading up-patterns and leading bottom-patterns, respectively. Finally, the correlation scores were ranked and used for predicting cancer types.

## Results

### An overview of eTumorType

Eighteen common cancer types containing 5327 samples from TCGA were included in this study (Table 1). CNVs of the founding clones were generated using the SNP 6.0 data of tumors and software tools including GISTIC 2.0 and ABSOLUTE (see Method). To properly construct and validate eTumorType, we split the whole dataset into the training, validation, and test sets, with the proportions of 60%, 20%, and 20%, respectively. To develop an algorithm (eTumorType) that is able to predict cancer types based on CNVs of the founding clones, we took a cancer hallmark approach, because cancer hallmarks are able to capture the most important genes that are closely related to cancer biology [23]. This approach has been successfully used to identify high accurate and robust gene expression-based biomarkers for breast and colon cancers [13], [14], [19], [24]. In addition, we focused on the modeling of CNVs of tumor founding clones in eTumorType.

As shown in Figure 1, we used cancer hallmark-associated GO terms composed of discriminating amplified genes to construct predictive models that are able to discriminate a pair of cancer types. Twelve GO terms were selected based on six cancer hallmarks (see Methods) to generate GO-ada models on the training set for each pair of cancer types (Figure 1). For a given sample, all the GO-ada models were used to vote which cancer types that sample could belong to. Then, we counted the number of votes for each cancer type (*i.e.*, voting profile). For a given cancer type, we averaged the voting numbers of each cancer type for the voting profiles of all the samples of that cancer type. We found that the average numbers of GO-ada model voting for cancer types were very similar among the training, validation, and test sets for each cancer type (Figures 2; S1–S4). To show this stability, the centroid of the average numbers of GO-ada model voting for each cancer type across samples for a cancer type was evaluated for each possible cancer type and then compared among the three datasets (see Methods). The results showed very similar centroid patterns, for example, 16 out of 18 cancer types had high correlation coefficients (>0.96), while LAML and skin cutaneous melanoma (SKCM) got relatively lower correlations of 0.93 and 0.89 (Table 2). These results indicate that predicting cancer types using founding clone CNVs of cancer hallmark-associated genes performs stably across different sample populations, and therefore contributes to robustness.

### Predictions using eTumorType

We first conducted a centroid-based correlation prediction (see Method) for the samples in the validation and test sets. A sample could be predicted to a set of cancer types (see Method). When considering the top-1 cancer type alone, only five cancer types were observed with accuracy of ≥0.80 in the validation set, but none in the test set (Table 3). When considering the 3 top-ranked cancer types, the prediction accuracies were improved greatly (Table 4). For example, the accuracy and power (recall rate) for breast invasive carcinoma, luminal subtype (LUMINAL) samples were 0.81 and 0.55 for the validation set when choosing the top-1 cancer type (Table 3), whereas they were respectively improved to 0.93 and 0.73 when choosing the 1–3 top-ranked cancer types (Table 4). For stomach adenocarcinoma (STAD) prediction, the accuracy and power were dramatically improved from 0.62 and 0.51 to 0.81 and 0.63, respectively (Table 3, Table 4). These results suggest that selecting the top-3 cancer type candidates would lead to a more reliable diagnosis, and more importantly, considering multiple possibilities can also be beneficial to patients as it is still able to guide examinations and would lead to detect the cancer with high probability. Of note, the interpretation of the prediction results is that a sample is most likely to be any of the three cancer types. However, clinically it is useful, as the predictions allow furthering checking only three cancer sites. For example, the CNVs identified by sequencing of CTCs from a patient (the cancer type is unknown, but the CTCs are captured in blood) could be used for predicting three possible cancer sites using eTumorType. Clinicians could further check the patients to find out which of the three cancers could be. This will be time-saving when searching the cancer sites.

The centroid-based correlation prediction showed that some cancer types had similarity, leading to the failure of ranking in top 1, which could be taken into account to shrink down the cancer type possibilities and then able to improve prediction. For example, the LUSC samples got higher numbers of GO-ada models voting for OV, LUAD, HNSC, and LUSC, but very lower votes for THCA and LAML (Figure 2). This result indicates that the LUSC samples have higher chance to be predicted as the former four cancer types, but lower chance to be predicted as the latter two cancer types. Therefore, if these two leading patterns were given weights, the prediction accuracy might be boosted.

By applying the leading pattern-weighted correlation prediction, the prediction performance was significantly improved (Table 5). For some cancer types, both prediction accuracy and power were increased. For example, in the validation set, for colon adenocarcinoma/rectum adenocarcinoma (COAD/READ), the accuracy and power of the prediction was enhanced from 0.92 to 0.94 and from 0.79 to 0.88, respectively; similar results were obtained for the test set (Table 4, Table 5). In addition, similar results were observed for glioblastoma multiforme (GBM), THCA, STAD, and kidney renal clear cell carcinoma (KIRC). On the other hand, for some other cancer types, the predictions were increased in either accuracy or power only. For example, the prediction for LAML in the validation set was improved from 0.81 to 0.92 for accuracy but decreased from 0.81 to 0.69 for power, with similar observations noticed in the test set. Usually, the trade-off between accuracy and power is inherent in prediction models, like the prediction for LAML. The improvements on both indices for COAD/READ suggest that the leading pattern-weighted correlation prediction works very well for this cancer type. That is, the selected leading patterns accurately evaluated its similarity and dissimilarity to the other cancer types in the leading up-patterns and bottom-patterns, thereby working efficiently in the prediction for true samples. On the other hand, the increase in accuracy but decrease in power for LAML indicates that the leading patterns enhanced the ability of filtering false samples but lowered down the competing power against cancer types sharing similarity with resulting in losing more true samples.

In summary, the prediction performances for the cancer types of COAD/READ, GBM, brain lower grade glioma (LGG), prostate adenocarcinoma (PRAD), and KIRC were increased significantly, from 0.85 to 0.92 and from 0.83 to 0.87 on average for accuracy and power, respectively, in the training set. The corresponding increases in the test set were from 0.81 to 0.90 and from 0.82 to 0.86 on average for accuracy and power, respectively. In the validation set, the prediction performances for the cancer types of OV, LUMINAL, LUAD, LUSC, uterine corpus endometrial carcinoma (UCEC), STAD, HNSC, LAML, and SKCM were increased moderately, from 0.80 to 0.84 and from 0.74 to 0.79 on average for accuracy and power, respectively. For the test set, the accuracy and power on average were from 0.77 to 0.83 and from 0.72 to 0.76. In total, these cancer types accounted for 14 of 18 types analyzed in this study. Therefore, the leading pattern-weighted correlation is able to discriminate cancer types.

## Discussion

eTumorType integrates the traits favoring robustness including cancer hallmarks composed of DAGs, stochastic algorithm of ada, voting mechanism based on the screening of cancer type comparisons, and a further centroid generation. For a given cancer type, the centroids of average number of GO-ada voting for each cancer type were stable across the training, validation, and test sets. In general, eTumorType performs well in the predictions. The current predictive models have been constructed based on the 18 common cancer types, because they have relatively larger sample sizes in TCGA. For other cancer types, when their sample numbers get large enough, we will refine our models to include them in the future. This is the limitation of the current predictive models. However, eTumorType is capable of discriminating cancer types, which is beyond the current studies on CTC or cfDNA data, as the majority of these studies only focused on finding features (*e.g.*, CNV, mutation, microRNA, methylation, and gene expression) associated with specific cancer types [25], [26], [27], [28], [29], [30]. To our best knowledge, no such algorithms have been developed so far. Our algorithm enables discriminating cancer types for CTCs and cfDNAs, which will be useful in early diagnosis of cancers in the near future.

The number of input genes affects the performance of GO-ada voting. When using the top-30 genes, the predictions for OV, LUAD, COAD/READ, GBM, KIRC, HNSC, and LAML samples had relatively good performance with the accuracy of 0.87 and power of 0.81 on average in the validation set and the corresponding values in the test set were 0.86 and 0.76. On average, the leading pattern-weighted correlation method led to prediction accuracy and power of 0.78 and 0.73 for all cancer types in both the validation and the test sets, which were around 4% and 6% less than the predictions using the top-100 genes.

In general, inclusion of the leading patterns improved the prediction. However, there was trade-off between accuracy and power when predicting some cancer types. This could be attributed to the inaccurate selection of the leading patterns. In our future work, we will improve the accuracy of selection and investigate the weights of the leading patterns in favor of reducing the trade-off.

Previously, we suggested that genomics and systems biology research should be conducted at sub-clonal and founding clonal levels [31], [32]. In this study, the founding clone CNVs were used not only for facilitating a smooth application of the method to CTC and cfDNA data, but also for getting insight for the early detection of cancer types. The formation of the founding clone represents cancer occurrence and cancer develops faster thereafter [9]. Therefore, signals derived from sub-clones cannot be much helpful for early detection.

Many studies have focused on somatic mutation-based biomarker discovery for cancer detection. However, somatic CNVs may have advantages as they have strong signals [12], whereas cancer genomes have rare somatic mutations, which are not common between tumor samples of even a same cancer type [33], [34]. In the meantime, the cost is much lower for CNV detection than mutation detection by genome sequencing, leading to the increasing tendency of measuring genome-level CNVs. Therefore, using CNVs could be a reliable and feasible option for early detection of cancer. The non-invasive manner of CTC capturing is a further advance for early cancer detection. Our eTumorType for discriminating cancer types is developed to fulfill this task by reliably identifying cancer type candidates. The good performance of the method for majority of the 18 common cancer types in this study holds promise for it.

Non-invasive cancer biomarker discovery has been studied on CTCs and cfDNAs in blood, serum, and plasma. Studies have reported similarity of CNVs in CTCs and cfDNAs with primary tumors [35], [36], [37], [38]. These results support the applicability of eTumorType on CTC and cfDNA data. Nevertheless, there are issues needed to be considered. First, the rate of false negative of CNV detection is large [5], [35], which might not detect enough DAGs to reach a good performance. Second, isolating rare CTCs and cfDNAs is challenging, especially for the early detection of cancer. Moreover, the necessary step of amplifying rare CTCs and cfDNAs makes it worse because biases and errors might be introduced [6], [39]. Furthermore, there are analyses showing the discordance between CTCs and primary tumors. For example, 48% of 62 metastatic breast cancers were reported discrepant between CTCs and the primary tumors on gene expression of 35 CTC-specific genes; 24% of patients had discrepant expression of the estrogen receptor [40]. The difference in signal intensities for genes might also indicate the discrepancy of CNVs between CTCs and primary tumors even for those detected as aberrations in both materials. Due to the inconsistent conclusions, searching biomarkers or models for early detection should be ideally performed based on CTCs and cfDNAs. However, this needs improved technologies and large datasets, which could be available in the future. Our study is likely to be a simulation for this task by comprehensively considering the founding clone CNVs and possibly small number of CNVs. The single-cell sequencing technology is promising to increase the resolution and make cancer genome sequencing data cleaner, which might make it easier to analyze the data. With the development of the related technologies, we believe that non-invasive cancer diagnosis would be accomplished in the near future.

## Conclusion

In this study, we developed eTumorType, which enables the identification of cancer types for CTCs or cfDNAs in blood, based on CNVs in the tumor founding clone. This application could be used for the early detection of 18 common cancers. The approach of using the tumor founding clone, in which genomic changes will be carried onto every cancer cells of the tumor, helps us to capture the most important genomic variations for that tumor. The cancer hallmarks considered in eTumorType allow capturing the genomic variations which are most likely to be associated with cancer development and progression, thereby contributing to robustness. Furthermore, to improve the prediction performance of eTumorType in clinical diagnosis, we developed a leading pattern-correlation method, which increased the prediction accuracy and power. The analysis performed in 5327 tumor samples of 18 cancer types provides a reliable evaluation of the algorithm. We hope eTumorType prediction would shed light on non-invasive early diagnosis of cancer types in the future.

## Authors’ contributions

EW conceived the idea and supervised the study. EW and JZ designed the algorithm. JZ implemented the algorithm and performed the analysis. JZ and EW analyzed the results. JZ drafted the manuscript and EW edited the manuscript. Both authors read and approved the final manuscript.

## Competing interests

The authors have declared that no competing interests exist.

## Figures and Tables

**Table 1:** Cancer types and sample sizes in the somatic founding clone CNV profile

**Cancer**	**Abbreviation**	**No. of samples**
Ovarian serous cystadenocarcinoma	OV	538
Breast invasive carcinoma (luminal subtype)	LUMINAL	531
Colon adenocarcinoma/rectum adenocarcinoma	COAD/READ	513
Glioblastoma multiforme	GBM	467
Kidney renal clear cell carcinoma	KIRC	415
Lung squamous cell carcinoma	LUSC	403
Uterine corpus endometrial carcinoma	UCEC	401
Lung adenocarcinoma	LUAD	395
Head and neck squamous cell carcinoma	HNSC	335
Brain lower grade glioma	LGG	244
Thyroid carcinoma	THCA	203
Stomach adenocarcinoma	STAD	177
Bladder urothelial carcinoma	BLCA	151
Prostate adenocarcinoma	PRAD	149
Cervical squamous cell carcinoma and endocervical adenocarcinoma	CESC	149
Breast invasive carcinoma (basal subtype)	BASAL	91
Skin cutaneous melanoma	SKCM	83
Acute myeloid leukemia	LAML	82

*Note*: CNV, copy number variation.

**Table 2:** Correlation coefficients of centroids among the training, validation, and test sets for various cancer types

**Cancer**	**Training *vs.* validation**	**Training *vs.* test**	**Validation *vs.* test**	**Minimum**
LUSC	1	1	1	1
LUMINAL	0.99	0.99	0.99	0.99
GBM	0.99	1	1	0.99
KIRC	1	0.99	0.99	0.99
LGG	0.99	0.99	1	0.99
OV	1	0.99	0.99	0.99
THCA	0.99	0.99	0.99	0.99
COAD/READ	0.99	0.99	0.98	0.98
HNSC	0.98	0.99	0.98	0.98
UCEC	0.99	0.98	0.99	0.98
CESC	0.97	0.97	0.99	0.97
LUAD	0.99	0.99	0.97	0.97
PRAD	0.97	0.98	0.97	0.97
BLCA	0.97	0.96	0.98	0.96
BASAL	0.96	0.96	0.98	0.96
STAD	0.96	0.97	0.97	0.96
LAML	0.97	0.93	0.97	0.93
SKCM	0.92	0.89	0.96	0.89

*Note*: The abbreviations of cancers are explained in Table 1.

**Table 3:** Accuracy and power of centroid-based cancer type prediction using top-1 selections

**Cancer**	**Training set**		**Validation set**		**Test set**	
	**Accuracy**	**Power**	**Accuracy**	**Power**	**Accuracy**	**Power**
OV	0.76	0.76	0.82	0.89	0.72	0.72
LUMINAL	0.73	0.51	0.81	0.55	0.60	0.42
LUAD	0.66	0.56	0.75	0.68	0.65	0.42
LUSC	0.59	0.82	0.67	0.91	0.51	0.73
COAD/READ	0.79	0.59	0.89	0.70	0.66	0.46
GBM	0.77	0.81	0.86	0.89	0.74	0.81
UCEC	0.46	0.10	0.70	0.35	0.40	0.13
THCA	0.29	0.86	0.35	0.85	0.27	0.93
STAD	0.53	0.43	0.62	0.51	0.38	0.39
LGG	0.71	0.64	0.73	0.71	0.46	0.47
PRAD	0.62	0.71	0.69	0.73	0.39	0.50
KIRC	0.74	0.49	0.80	0.64	0.73	0.39
HNSC	0.57	0.49	0.61	0.64	0.36	0.36
CESC	0.50	0.73	0.51	0.63	0.28	0.43
LAML	0.66	0.98	0.71	0.75	0.44	0.41
BLCA	0.62	0.56	0.54	0.47	0.39	0.23
BASAL	0.33	0.93	0.34	0.72	0.30	0.72
SKCM	0.52	0.74	0.47	0.41	0.36	0.50
Average	0.60	0.65	0.66	0.67	0.48	0.50

*Note*: The abbreviations of cancers are explained in Table 1.

**Table 4:** Accuracy and power of centroid-based cancer type prediction using top-3 selections

**Cancer**	**Training set**		**Validation set**		**Test set**	
	**Accuracy**	**Power**	**Accuracy**	**Power**	**Accuracy**	**Power**
OV	0.84	0.93	0.86	0.94	0.78	0.92
LUMINAL	0.93	0.80	0.93	0.73	0.88	0.66
LUAD	0.89	0.76	0.81	0.81	0.90	0.59
LUSC	0.82	0.98	0.76	0.98	0.77	0.95
COAD/READ	0.93	0.83	0.92	0.79	0.83	0.65
GBM	0.94	0.90	0.90	0.91	0.89	0.87
UCEC	0.92	0.35	0.85	0.43	0.78	0.50
THCA	0.48	0.93	0.46	0.88	0.46	0.98
STAD	0.90	0.72	0.81	0.63	0.66	0.64
LGG	0.91	0.94	0.78	0.82	0.77	0.88
PRAD	0.78	0.93	0.76	0.83	0.68	0.93
KIRC	0.91	0.92	0.87	0.80	0.86	0.77
HNSC	0.86	0.85	0.71	0.78	0.68	0.75
CESC	0.74	0.91	0.65	0.80	0.63	0.73
LAML	0.83	0.98	0.81	0.81	0.81	0.76
BLCA	0.80	0.81	0.74	0.57	0.65	0.43
BASAL	0.59	1.00	0.48	0.83	0.52	0.89
SKCM	0.85	0.94	0.67	0.59	0.67	0.75
Average	0.83	0.86	0.77	0.77	0.73	0.76

*Note*: The abbreviations of cancers are explained in Table 1.

**Table 5:** Accuracy and power of leading pattern-weighted correlation prediction of caner types using top-3 selections

**Cancer**	**Training set**		**Validation set**		**Test set**	
	**Accuracy**	**Power**	**Accuracy**	**Power**	**Accuracy**	**Power**
OV	0.90	0.94	0.83	0.93	0.80	0.93
LUMINAL	0.93	0.97	0.85	0.88	0.83	0.85
LUAD	0.96	0.86	0.91	0.87	0.82	0.70
LUSC	0.85	0.98	0.80	0.96	0.78	0.95
COAD/READ	0.96	0.95	0.94	0.88	0.87	0.79
GBM	0.97	0.91	0.92	0.92	0.92	0.89
UCEC	0.94	0.73	0.81	0.58	0.84	0.66
THCA	0.80	0.98	0.62	0.90	0.56	0.98
STAD	0.98	0.89	0.81	0.71	0.80	0.67
LGG	0.97	0.97	0.91	0.82	0.90	0.88
PRAD	0.94	0.94	0.93	0.87	0.96	0.87
KIRC	0.96	0.98	0.91	0.84	0.86	0.88
HNSC	0.93	0.90	0.81	0.91	0.80	0.76
CESC	0.78	0.93	0.67	0.73	0.65	0.73
LAML	1.00	0.96	0.92	0.69	0.91	0.59
BLCA	0.93	0.89	0.76	0.63	0.71	0.40
BASAL	0.90	0.96	0.67	0.67	0.70	0.78
SKCM	0.94	0.94	0.83	0.59	0.92	0.69
Average	0.92	0.93	0.83	0.80	0.81	0.78

*Note*: The abbreviations of cancers are explained in Table 1.

## Supplementary material

Supplementary Table S1 Leading patterns used in this study based on selecting 100 top-ranked DAGs for each cancer hallmark GO term
