# Supplementary material for: eTumorType, An Algorithm of Discriminating Cancer Types for Circulating Tumor Cells or Cell-free DNAs in Blood
Source: Genomics Proteomics Bioinformatics. 2017 Apr 4;15(2):130–40. doi: 10.1016/j.gpb.2017.01.004 (PMC5414714; doi:10.1016/j.gpb.2017.01.004)
Supplement: Supplementary Table S1 — Leading patterns used in this study based on selecting 100 top-ranked DAGs for each cancer hallmark GO term [file mmc1.docx]

**Table S1 Leading patterns used in this study based on selecting 30–100 top-ranked DAGs for each cancer hallmark GO term**

| **Cancer** | **Leading up-pattern** | **Leading bottom-pattern** |
| --- | --- | --- |
| BLCA | BLCA, LUAD, UCEC, STAD, HNSC | LAML, THCA |
| BASAL | BASAL, OV, UCEC | THCA, LAML |
| LUMINAL | LUMINAL, LUAD, UCEC | LAML, SKCM |
| CESC | CESC, HNSC, LUSC, UCEC | BASAL, LAML |
| COAD/READ | COAD/READ, STAD, UCEC | BASAL, LAML |
| GBM | GBM, LGG, KIRC | BASAL, LAML |
| HNSC | HNSC, LUSC, LUAD, UCEC, CESC | BASAL, LAML |
| KIRC | KIRC, LUAD, UCEC | BASAL, OV, LAML |
| LAML | LAML, UCEC, LGG, KIRC | BASAL, LUSC |
| LGG | LGG, UCEC, GBM, KIRC | BASAL, LUSC, CESC |
| LUAD | LUAD, LUMINAL, UCEC, STAD, BLCA | LAML, THCA |
| LUSC | LUSC, HNSC, OV, LUAD | THCA, LAML |
| OV | OV, UCEC, LUSC, HNSC | LAML, THCA |
| PRAD | PRAD, UCEC, LGG, KIRC | BASAL, OV, LUSC, CESC, LAML |
| SKCM | SKCM, LUMINAL, LUAD, COAD/READ | BASAL, LAML |
| STAD | STAD, COAD/READ, UCEC | THCA, LAML, BASAL |
| THCA | THCA, UCEC, KIRC | BASAL, LUSC |
| UCEC | UCEC, LUMINAL, LUAD | BASAL, LAML, SKCM |

*Note*: The abbreviations of cancers are explained in Table 1. Leading up- and bottom-pattern represent the most similar and the most dissimilar cancer type groups to the given cancer type, respectively. DAG, differentially-amplified gene; GO, gene ontology.
